# Association between periconceptional weight loss and maternal and neonatal outcomes in obese infertile women

**DOI:** 10.1371/journal.pone.0192670

**Published:** 2018-03-28

**Authors:** Anne M. van Oers, Meike A. Q. Mutsaerts, Jan M. Burggraaff, Walter K. H. Kuchenbecker, Denise A. M. Perquin, Carolien A. M. Koks, Ron van Golde, Eugenie M. Kaaijk, Frank J. Broekmans, Jan Peter de Bruin, Fulco van der Veen, Annemiek W. Nap, Ed T. C. M. Gondrie, Ben W. J. Mol, Henk Groen, Annemieke Hoek, on behalf of the LIFEstyle study group

**Affiliations:** 1 Department of Obstetrics and Gynecology, University Medical Center Groningen, University of Groningen, Groningen, The Netherlands; 2 Department of General Practice, University Medical Centre Utrecht, University of Utrecht, Utrecht, The Netherlands; 3 Department of Obstetrics and Gynecology, Scheper Hospital, Emmen, The Netherlands; 4 Department of Obstetrics and Gynecology, Isala, Zwolle, The Netherlands; 5 Department of Obstetrics and Gynecology, Medical Centre Leeuwarden, Leeuwarden, The Netherlands; 6 Department of Obstetrics and Gynecology, Máxima Medical Centre, Veldhoven, The Netherlands; 7 Department of Obstetrics and Gynecology, Maastricht University Medical Centre, University of Maastricht, Maastricht, the Netherlands; 8 Department of Obstetrics and Gynecology, OLVG, Amsterdam, The Netherlands; 9 Department for Reproductive Medicine, Division Female and Baby, University Medical Centre Utrecht, University of Utrecht, Utrecht, The Netherlands; 10 Department of Obstetrics and Gynecology, Jeroen Bosch Hospital, ‘s-Hertogenbosch, The Netherlands; 11 Centre for Reproductive Medicine, Academic Medical Centre, University of Amsterdam, Amsterdam, The Netherlands; 12 Department of Obstetrics and Gynecology, Rijnstate, Arnhem, The Netherlands; 13 Department of Obstetrics and Gynecology, Zuyderland MC, Sittard, The Netherlands; 14 School of Paediatrics and Reproductive Health, The Robinson Research Institute, University of Adelaide, Adelaide, Australia; 15 Department of Epidemiology, University Medical Centre Groningen, University of Groningen, Groningen, The Netherlands; University of Tennessee Health Science Center, UNITED STATES

## Abstract

**Background:**

Obesity in women of reproductive age has deleterious effects on reproductive and offspring health. In this study, we aimed to evaluate the association between the magnitude of periconceptional body-mass index (BMI) change and maternal and neonatal outcomes in obese infertile women who participated in the LIFEstyle study. The LIFEstyle study was a randomized controlled trial, evaluating if a six-month lifestyle intervention program prior to infertility treatment in obese infertile women improved birth rates, compared to prompt infertility treatment.

**Methods and findings:**

This is an exploratory post hoc analysis of the LIFEstyle study. We recorded periconceptional BMI change in women with an ongoing pregnancy, pooling data of all women, regardless of randomization arm. Periconceptional BMI change was calculated using weight at randomization and the periconceptional weight (measured in kilograms 12 weeks before or after conception and expressed as BMI change in units BMI (kg/m^2^)). Subsequently, women were categorized into quartiles according to the magnitude of their periconceptional change in BMI. The odds of maternal and neonatal outcomes were calculated using logistic regression analysis, comparing women in each of the first three weight change quartiles separately, and combined, to women in the fourth quartile. The fourth quartile was chosen as reference group, since these women had the least weight loss. We adjusted for periconceptional BMI, nulliparity and smoking status. In addition, we performed a subgroup analysis for singleton pregnancies. In the LIFEstyle study, 321 obese infertile women achieved an ongoing pregnancy which was conceived within 24 months after randomization. Periconceptional BMI change was available in 244 of these women (76%). Median BMI at randomization was 35.9 kg/m^2^. Women in the first quartile (Q1) had a periconceptional BMI change of <-2.1 kg/m^2^, women in the second quartile (Q2) -2.1 to -0.9 kg/m^2^, women in the third quartile (Q3) -0.9 to 0.1 kg/m^2^ and women in the fourth quartile (Q4) gained ≥0.1 kg/m^2^. There were no significant differences between women in the quartiles regarding rates of excessive gestational weight gain (in term pregnancies), gestational diabetes, preterm birth, induction of labor, spontaneous vaginal birth and Caesarean section. Compared to women in Q4, the adjusted odds ratios, aOR, and 95% confidence interval for a hypertensive complication were; 0.55 (0.22–1.42) for women in Q1, 0.30 (0.12–0.78) for women in Q2, 0.39 (0.16–0.96) for women in Q3 and 0.39 (0.19–0.82) for women in Q1 to Q3 combined. In the subgroup analysis, investigating singleton pregnancies only, the statistically significant decreased rate of a hypertensive complication remained in women in Q2 (aOR 0.27, 95% CI 0.10–0.72) and Q3 (aOR 0.39, 95%CI 0.16–0.98) and when comparing women in Q1 to Q3 together to women in Q4 (aOR 0.38, 95%CI 0.18–0.80). Furthermore, there was a significantly decreased aOR (95%CI) of preterm birth in women in Q2 (0.24, 0.06–0.98) and when combining women in Q1 to Q3 (0.37, 0.14–0.97) compared to women in Q4.

**Conclusions:**

These results suggest that a periconceptional decrease in BMI in obese infertile women could lead to a decrease of the rates of hypertensive pregnancy complications and preterm birth. The results are limited by the exploratory nature of the analyses and further evidence is necessary to provide more definitive conclusions.

## Introduction

Obesity, defined as a body-mass index (BMI) ≥30kg/m^2^, has deleterious effects on women’s reproductive health and health of their offspring [1–4]. Obese women are at an increased risk of adverse pregnancy outcomes, such as gestational hypertension, preeclampsia and gestational diabetes [5,6]. Moreover, there is an increased risk of induction of labor, shoulder dystocia, postpartum hemorrhage and Caesarean section [5,7,8]. Maternal obesity is also associated with an increased risk of adverse neonatal outcomes, including an increased rate of preterm birth, large-for-gestational age (LGA) infants, neonatal intensive care unit (NICU) admission, congenital anomalies and perinatal death [9–12]. Furthermore, children born from obese mothers have an increased risk of childhood and adulthood overweight or obesity and increased mortality rate later in life [3,13–15].

Studies investigating the effect of antenatal lifestyle interventions to prevent excessive gestational weight gain and/or obesity-associated complications in overweight and obese pregnant women report limited clinical risk reduction of maternal and perinatal complications [16–20]. Preconceptional interventions might have a greater impact on the reduction of obesity-associated complications during pregnancy and in offspring, as is exemplified by studies on the effects of preconceptional bariatric surgery [21,22]. Even though rates of macrosomia and large-for-gestational age (LGA) are generally decreased after bariatric surgery, rates of small-for-gestational age (SGA) are increased [22,23].

We conducted a randomized controlled trial (RCT), in which we investigated reproductive outcomes in obese infertile women who were randomized to a six-month lifestyle intervention program preceding infertility treatment, as compared to prompt infertility treatment [24]. The lifestyle intervention did not result in higher rates of the primary outcome, the vaginal births of a healthy singleton at term within 24 months after randomization, while it did result in comparable rates of ongoing pregnancies and more natural conceptions in the intervention group [25]. Rates of maternal and neonatal complications were not different between randomization groups, but the influence of the magnitude of weight loss on these outcomes was not analyzed separately.

Only few studies have been performed regarding the association between periconceptional weight loss and maternal and neonatal outcomes in obese women [26]. It is not known whether a periconceptional decrease in BMI improves pregnancy outcomes in women who did not receive bariatric surgery and what amount of weight loss could do so. Therefore, the purpose of the current analysis is to investigate whether periconceptional weight loss influences maternal and neonatal pregnancy outcomes and to determine what amount of weight loss could affect these outcomes.

## Materials and methods

We used data from the LIFEstyle study, a multicenter RCT. The study protocol and results of the LIFEstyle study have been reported previously [24,25]. The study protocol of the LIFEstyle study was approved by the Medical Ethics Committee (MEC; 2008.284) of the University Medical Centre Groningen and the board of directors of each participating center. The study was conducted in adherence to the Declaration of Helsinki and all participants gave written informed consent. In short, obese infertile women were randomly allocated to a lifestyle intervention program preceding infertility treatment (intervention group) or to prompt infertility treatment (control group). Women in the intervention group received a six-month lifestyle intervention, which consisted of an energy-restricted diet, an increase of physical activity and motivational counseling. The lifestyle intervention was aimed at 5–10% weight loss and the program included six outpatient visits and four telephone consultations. After completion of the six-month lifestyle intervention, women could commence with infertility treatment if they had not conceived naturally during the intervention period. Women in the control group started infertility treatment promptly after randomization. Infertility treatment in both study arms could consist of expectant management, ovulation induction, intrauterine insemination, in vitro fertilization or intracytoplasmic sperm injection including transfers of frozen embryos, depending on the diagnosis. The indicated type of treatment was based on guidelines from the Dutch Society of Obstetrics and Gynecologists [27]. Women were followed for 24 months after randomization. Data on the course of pregnancy and childbirth was also recorded when a woman conceived within 24 months after randomization but childbirth occurred after the 24 months of follow-up. The primary outcome of the LIFEstyle study was the vaginal birth of a healthy singleton at term within 24 months after randomization. A power calculation was performed using the primary outcome of the study [24]. A total of 63 women (21.8%) discontinued the lifestyle intervention. The mean weight loss at 6 months after randomization was 4.4kg in 236 non-pregnant women in the intervention group and 1.1 kg in 128 non-pregnant women in the control group (P<0.001)[25].

### Periconceptional weight change

For the current analysis we used pooled data, regardless of randomization arm, of women who had an ongoing pregnancy and complete follow-up during the LIFEstyle study. By doing so, we aimed to investigate the association between pregnancy outcomes and periconceptional change in BMI, irrespective of its cause, be it participation in the intervention or possibly a personal initiative to change diet after receiving the information of the deleterious effects of overweight as part of the patient information provided at the start of the study. Furthermore, this is an exploratory post-hoc analysis, which was not described in the study protocol prior to the start of the LIFEstyle study.

Women with an ongoing pregnancy that was conceived within 24 months after randomization, for whom data on periconceptional weight was available, were categorized into quartiles according to their change in BMI units (kg/m^2^). We used change in BMI since this accurately reflects change in body composition for women with different statures. We calculated weight change using weight at randomization and the periconceptional weight. Periconceptional weight was determined using the weight at the first antenatal visit (which for infertile couples is usually at 7 to 8 weeks gestation). When this was not available the self-measured weight at 12 weeks gestation or weight within 12 weeks of conception was used. To provide a clinical more meaningful outcome, women were also categorized in quartiles using periconceptional weight change in kilograms and the analyses were repeated.

### Maternal and neonatal outcomes

Maternal and neonatal outcomes were recorded for all ongoing pregnancies conceived within 24 months after randomization. Maternal adverse outcomes were: excessive gestational weight gain (>9 kilograms for women with BMI >30 kg/m^2^ or >11.5 kilograms for women with BMI 25–29.9kg/m^2^ in a term pregnancy) [28], gestational diabetes (any form of hyperglycemia during pregnancy) [29], hypertensive complications (pregnancy-induced hypertension [a systolic blood pressure of ≥ 140 mmHg and/or diastolic blood pressure of ≥ 90 mmHg at ≥ 20 weeks gestation, measured at two occasions], preeclampsia [pregnancy-induced hypertension with proteinuria of at least 300mg per 24 hours] and HELLP syndrome [characterized by hemolysis, elevated liver-enzyme levels, and a low platelet count]) [30]. In addition, we recorded preterm birth (<37 weeks gestational age), induction of labor (induction of labor by artificial means) and mode of delivery (spontaneous vaginal birth, assisted vaginal birth or Caesarean section).

Gestational weight gain was assessed using self-measured weight during pregnancy. Gestational weight gain records were considered complete when weight was measured from four weeks before parturition until the delivery in a term pregnancy and when the periconceptional BMI and weight in kilograms was known.

Neonatal outcomes were SGA or LGA (defined as birth weight below the 10th or above the 90th percentile according to the Dutch reference curves) [31]. Due to low prevalence of the individual complications a composite neonatal outcome was used, consisting of cord pH <7.05, Apgar score <7 at 5 minutes, admission to the NICU and perinatal death (stillbirth above 24 weeks gestation or early neonatal death within six weeks postpartum) [12]. For neonatal outcomes, the number of women was the denominator.

### Statistical analysis

Descriptive statistics are given as *n*, %, median and interquartile range (IQR) where appropriate. Baseline characteristics in the weight change quartiles were compared using the non-parametric Kruskall-Wallis test for continuous variables and the chi-square test for categorical variables.

The probabilities of maternal and neonatal outcomes were calculated as a function of weight change. For each outcome, we performed logistic regression analysis, comparing women in each of the first three weight change quartiles separately, and combined (women in Q1, Q2 and Q3 grouped together), to women in the fourth quartile. The fourth quartile was chosen as reference group, since these women had the least weight loss. P-values for linear relation of the aOR across the BMI change quartiles were calculated (using the quartiles as a continuous variable). We adjusted for periconceptional BMI, nulliparity and smoking status in the logistic regression analyses. Adjusted odds ratios (aOR) and 95% confidence intervals (CI) are presented. Since pregnancy of multiples affect maternal and neonatal outcomes, we repeated the analyses in singleton pregnancies. Randomization arm in the randomized controlled trial could be a potential confounder in the analysis, therefore logistic regression analyses with randomization group (intervention or control group), BMI change quartile and the interaction term between randomization group and quartile were performed without adjustment for confounders. A significant interaction between the BMI change quartiles and the lifestyle intervention was defined as a p-value <0.1.

All analyses were performed using Statistical Package for the Social Sciences Statistics, version 22 (IBM Corporation, Armonk, USA). Statistical significance was assumed with a p<0.05 (except for the analysis investigating statistical interaction).

## Results

**Fig 1:**
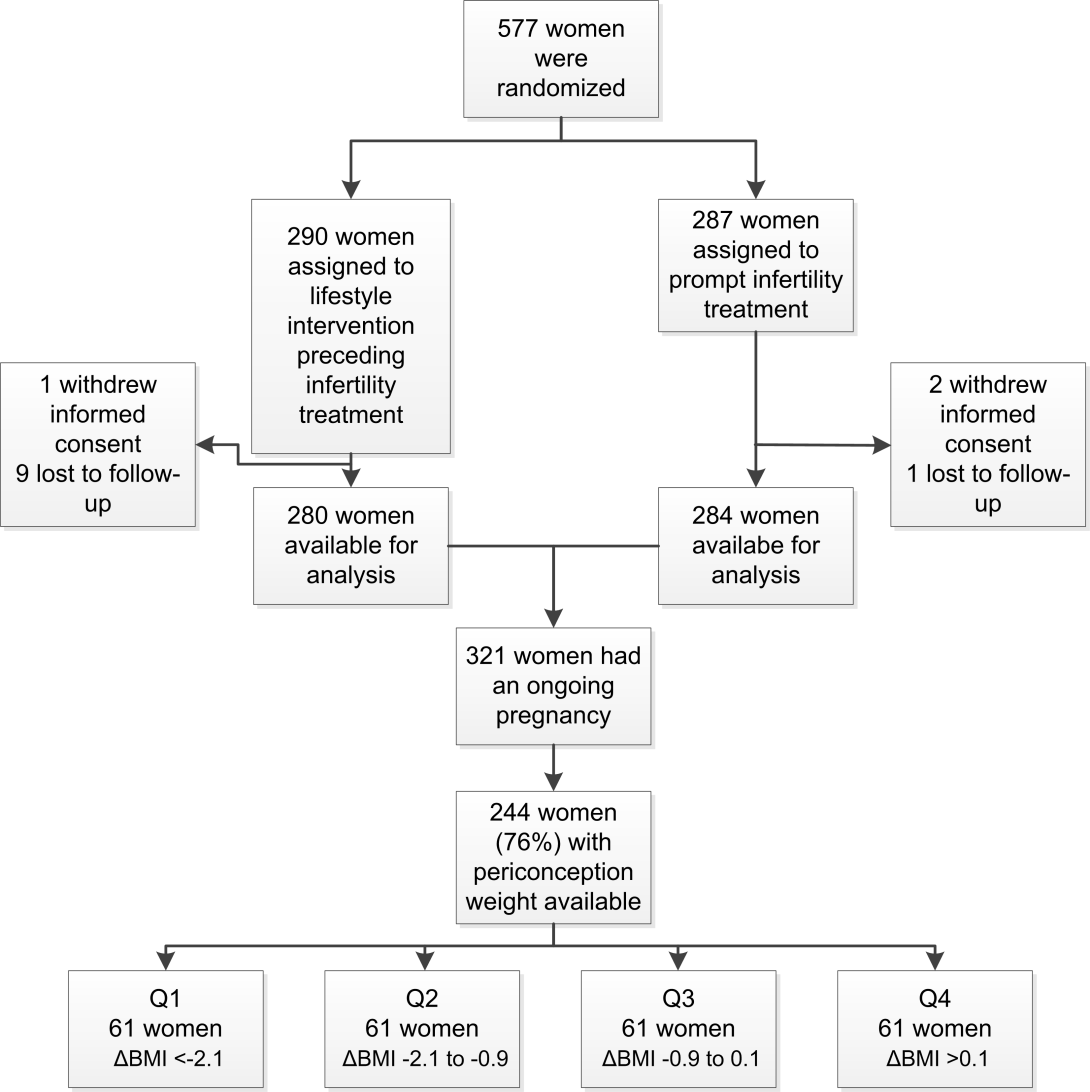
Flowchart of the analysis.

The flowchart of the analysis is shown in Fig 1. Of all 577 women who were randomized and provided informed consent, 290 women were allocated to the lifestyle intervention program preceding infertility treatment (intervention group) and 287 to prompt infertility treatment (control group). Ten women in the intervention group and three women in the control group had incomplete follow-up or withdrawal of informed consent. Of all women in the intervention and control group combined, 321 women conceived within 24 months after randomization and had an ongoing pregnancy. For 244 (76%) of these women the periconceptional weight change was available.

Median BMI at study entry was 36.0 kg/m^2^ (IQR 4.7 kg/m^2^). Median periconceptional BMI change in the 244 women was -0.9 kg/m^2^ (IQR -2.1 to 0.1 kg/m^2^). Women in the first quartile (Q1) had a periconceptional BMI change of <-2.1 kg/m^2^, women in the second quartile (Q2) -2.1 to -0.9 kg/m^2^, women in the third quartile (Q3) -0.9 to 0.1 kg/m^2^ and women in the fourth quartile (Q4) gained ≥0.1 kg/m^2^. Median weight at study entry was 103.8 kg (IQR 15.4 kg). Median periconceptional weight change in kilograms was -2.6 kg (IQR 6.5kg). Baseline characteristics are summarized in Table 1. Women who achieved most weight loss were more often randomized to the intervention group (p<0.01) and had a longer time to pregnancy (p<0.01). Women with a missing periconceptional weight had a significantly longer time to pregnancy compared to women with known periconceptional weight (p<0.01).

**Table 1:** Baseline characteristics of women according to weight change quartile.

		BMI change quartiles					All women included in the analysis	Women with missing weight change
	Quartile	Q1	Q2	Q3	Q4	p-value		
	ΔBMI (kg/m^2^)	<-2.1	-2.1 to -0.9	-0.9 to 0.1	>0.1			
	N	n = 61	n = 61	n = 61	n = 61		n = 244	n = 77
**Maternal characteristics at randomization**	unit							
Age	yr	30.0 (5.8)	30.5 (6.4)	28.6 (6.8)	28.6 (6.6)	0.44	29.3 (6.6)	27.9 (6.1)
Body-mass index	kg/m^2^	36.0 (3.3)	35.4 (6.1)	35.4 (5.5)	36.8 (3.8)	0.27	36.0 (4.7)	35.9 (5.1)
Blood pressure								
– Systolic	mmHg	122 (18)	125 (13)	122 (18)	124 (18)	0.74	125 (19)	129 (19)
– Diastolic	mmHg	80 (10)	80 (10)	80 (6)	80 (15)	0.96	80 (10)	80 (15)
Maternal smoking	N	12 (20)	10 (16)	11 (18)	13 (21)	0.87	46 (19)	22 (29)
Ethnic origin						0.93		
– Caucasian	n	55 (90)	54 (89)	55 (90)	53 (87)		217 (89)	68 (88)
– Other	n	6 (10)	7 (12)	6 (10)	8 (13)		27 (11)	9 (12)
Education						0.20		
– Primary school	n	2 (3.3)	1 (1.6)	3 (4.9)	2 (3.3)		8 (3.3)	2 (2.6)
– Secondary education	n	10 (16)	10 (16)	13 (21)	14 (23)		47 (19)	21 (27)
– Intermediate vocational education	n	26 (43)	27 (44)	33 (54)	33 (54)		119 (49)	33 (43)
– Higher vocational education and university	n	21 (34)	19 (31)	11 (18)	7 (12)		58 (24)	17 (22)
– Unknown	n	2 (3.3)	4 (6.6)	1 (1.6)	5 (8.2)		12 (4.9)	4 (5.2)
Randomized to lifestyle intervention	n	38 (62)	35 (57)	19 (31)	21 (34)	<0.01	113 (46)	41 (53)
Duration of infertility	months	21 (23)	17 (13)	19 (14)	19 (23)	0.23	19 (17)	16 (14)
Nulliparous	n	46 (75)	50 (82)	43 (71)	49 (80)	0.43	188 (77)	66 (86)
Time to pregnancy from randomization	months	7.8 (4.5)	4.4 (7.8)	3.3 (6.6)	5.2 (7.7)	<0.01	6.0 (7.1)	11 (10)^a^
Multiple pregnancy	n	5 (8.1)	1 (1.6)	0	2 (3.3)	0.07	8 (3.3)	8 (10)^a^
Periconceptional weight measured during						0.12		NA
– first antenatal visit	n	41 (67)	40 (66)	37 (61)	44 (72)		162 (66)	NA
– self-reported 12 weeks after gestation	n	11 (18)	13 (21)	9 (15)	14 (23)		47 (19)	NA
– measured within 12 weeks of conception	n	9 (15)	8 (13)	15 (25)	3 (4.9)		35 (14)	NA

Table shows number of women (%) or median (IQR) by quartiles of BMI change between randomization in the LIFEstyle study and the periconceptional period. Unless otherwise stated, baseline characteristics were measured at randomization. Medians were compared using the Kruskal-Wallis test and for categorical data using the Chi-square test.

^a^ Women with a missing periconceptional weight change had a significantly longer time to pregnancy, p<0.01 and more often a multiple pregnancy, p = 0.01.BMI, body-mass index NA, not applicable

**Fig 2:**
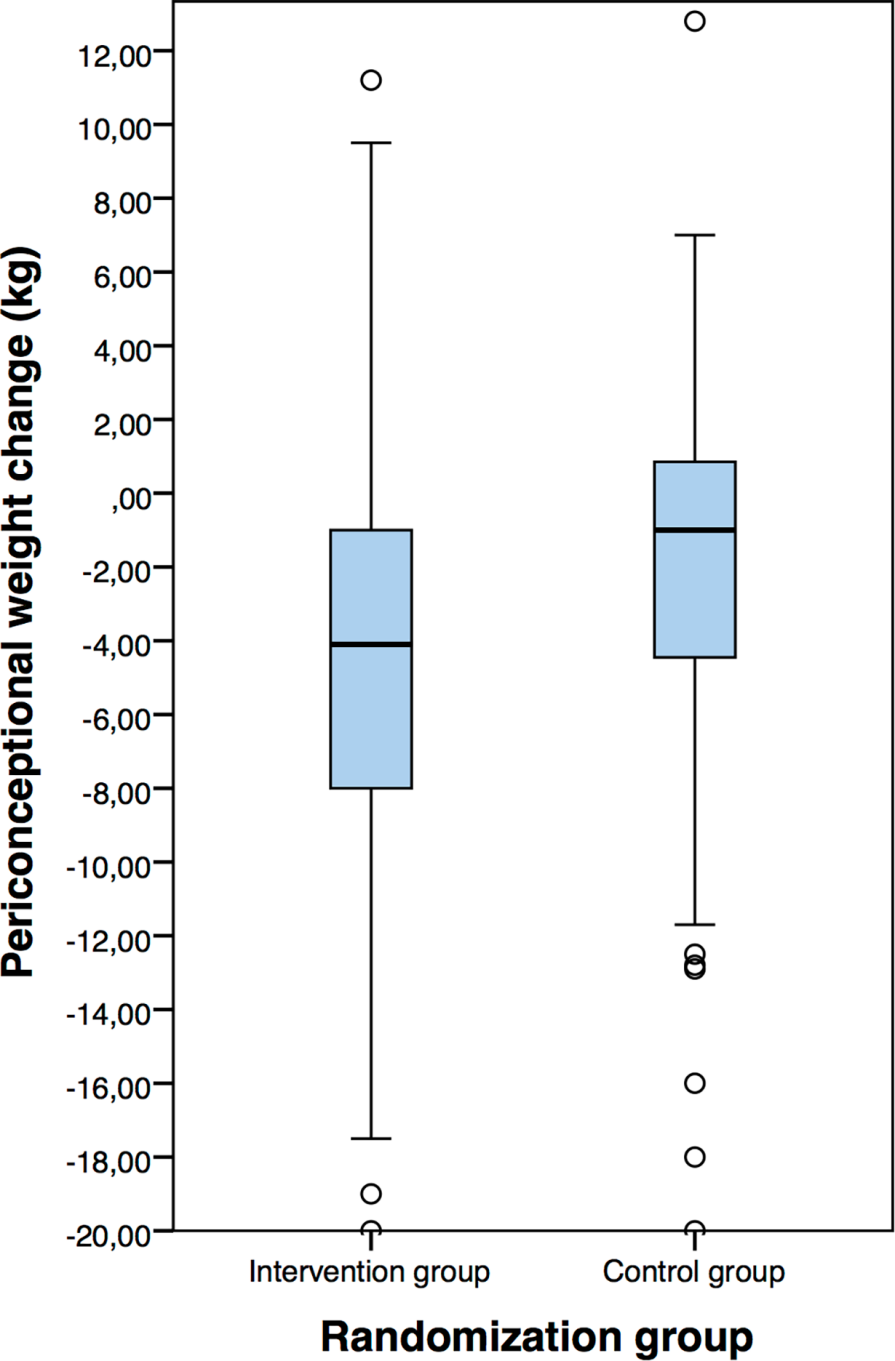
Periconceptional weight change in the intervention and control group.

Although periconceptional weight change in the intervention (-4.1 kg, IQR 7.2 kg) and control group (-1.0 kg, IQR 5.5 kg) was significantly different (p<0.001), there was a considerable overlap in periconceptional weight change (Fig 2).

### Maternal outcomes

There were no significant differences between quartile groups regarding rates of excessive gestational weight gain, gestational diabetes, preterm birth, induction of labor, spontaneous vaginal birth, assisted vaginal birth and Caesarean section (Table 2).

**Table 2:** Maternal outcomes by quartile of BMI change.

	Quartile	Q1	Q2	Q3	Q4		
	ΔBMI	<-2.1	-2.1 to -0.9	-0.9 to 0.1	>0.1	aOR Q1to 3 vs Q4^a^	P-value linear relation
**Rates of ongoing pregnancies within 24 months**							
		n = 61^b^	n = 61	n = 61	n = 61		
**Maternal outcomes**							
Excessive gestational weight gain^c^	rate (%)	21/34 (62)	20/30 (67)	10/26 (39)	14/27 (52)		
	aOR	1.71	2.06	0.61	1.00	1.17	0.15
	(95%CI)	(0.51–5.76)	(0.59–7.13)	(0.19–1.99)		(0.43–3.24)	
Gestational diabetes	rate (%)	8 (13)	8 (13)	15 (25)	10 (16)		
	aOR	0.93	0.93	1.69	1.00	1.24	0.61
	(95%CI)	(0.29–2.95)	(0.31–2.74)	(0.65–4.40)		(0.52–2.99)	
Hypertensive complications	rate (%)	14 (23)	9 (15)	10 (16)	21 (34)		
	aOR	0.55	0.30	0.39	1.00	0.39	0.17
	(95%CI)	(0.22–1.42)	(0.12–0.78)	(0.16–0.96)		(0.19–0.82)	
**Rates of live births conceived within 24 months**							
		n = 59	n = 60	n = 61	n = 60		
Preterm birth	rate (%)	10 (17)	4 (6.7)	5 (8.2)	12 (20)		
	aOR	0.88	0.30	0.37	1.00	0.46	0.75
	(95%CI)	(0.30–2.64)	(0.09–1.04)	(0.12–1.19)		(0.19–1.11)	
Induction of labor	rate (%)	25 (42)	20 (33)	25 (41)	30 (50)		
	aOR	0.92	0.59	0.76	1.00	0.73	0.71
	(95%CI)	(0.40–2.10)	(0.27–1.28)	(0.36–1.61)		(0.38–1.39)	
Spontaneous vaginal birth	rate (%)	33 (56)	44 (73)	36 (59)	33 (55)		
	aOR	0.76	1.91	0.95	1.00	1.15	0.94
	(95%CI)	(0.33–1.75)	(0.85–4.29)	(0.45–2.04)		(0.59–2.21)	
Assisted vaginal birth^d^	rate (%)	7 (18)	6 (12)	12 (25)	8 (20)		
	aOR	1.41	0.69	1.88	1.00	1.04	0.85
	(95%CI)	(0.38–5.22)	(0.20–2.33)	(0.63–5.58)		(0.69–1.56)	
Caesarean section	rate (%)	19 (32)	10 (17)	13 (21)	19 (32)		
	aOR	1.15	0.46	0.63	1.00	0.67	0.89
	(95%CI)	(0.47–2.81)	(0.19–1.16)	(0.27–1.48)		(0.33–1.36)	

Table shows rates and % of maternal outcomes by quartiles of BMI change. Odds ratios are adjusted for periconceptional BMI, nulliparity and smoking. P-values for the linear relation of quartiles of BMI change were calculated using the quartiles as a continuous variable, with adjustment for confounders.

^a^ Women in Q1, Q2 and Q3 were grouped together in the analysis and compared to women in Q4

^b^ One woman with an ongoing pregnancy had no follow-up during pregnancy and outcomes were not recorded

^c^ In term pregnancies only

^d^ The denominator is the total number of vaginal births

BMI, body-mass index, aOR, adjusted odds ratio, CI, confidence interval

A hypertensive complication occurred in 23% of women in Q1 (aOR 0.55, 95%CI 0.22–1.42), in 15% of women in Q2 (aOR 0.30, 95%CI 0.12–0.78), in 16% of women in Q3 (aOR 0.39, 95%CI 0.16–0.96) and 34% of women in Q4 (reference). When women in quartiles 1 to 3 were combined and compared to women in Q4 there was an aOR of 0.39 (95%CI 0.19–0.82) of having a hypertensive complication during pregnancy.

Maternal outcomes according to periconceptional weight change in kilograms are shown in S1 Table. Results were similar to the analysis using periconceptional change in BMI, except for the difference between hypertensive complications in women in Q3 compared to women in Q4, which was not significantly different (aOR 0.42, 95% CI 0.18–1.03).

### Neonatal outcomes

There were no significant differences in the rates of SGA, LGA or the composite adverse neonatal outcome among women within the BMI change quartiles (Table 3).

**Table 3:** Neonatal outcomes by quartile of BMI change.

	Quartile	Q1	Q2	Q3	Q4		
	ΔBMI	<-2.1	-2.1 to -0.9	-0.9 to 0.1	>0.1	aOR Q1 to3 vs Q4^a^	P-value linear relation
		n = 61^b^	n = 61	n = 61	n = 61		
**Maternal outcomes**							
SGA^c^	rate (%)	6 (10)	2 (3.3)	4 (6.6)	4 (6.7)		
	aOR	1.32	0.41	0.91	1.00	0.80	0.87
	(95%CI)	(0.27–6.44)	(0.07–2.56)	(0.20–4.13)		(0.21–3.03)	
LGA^c^	rate (%)	8 (14)	10 (17)	10 (16)	9 (15)		
	aOR	1.31	1.49	1.42	1.00	1.42	0.66
	(95%CI)	(0.41–4.19)	(0.52–4.27)	(0.51–3.99)		(0.58–3.50)	
Composite neonatal outcome	rate (%)	8 (13)	5 (8.2)	4 (6.6)	10 (16)		
	aOR	0.63	0.39	0.34	1.00	0.42	0.51
	(95%CI)	(0.19–2.08)	(0.11–1.33)	(0.10–1.20)		(0.16–1.12)	
Abnormal cord pH	rate (%)	1/40 (2.5)	1/35 (2.9)	1/37 (2.7)	1/39 (2.6)		
Apgar < 7	rate (%)	4 (6.7)	2 (3.3)	1 (1.6)	2 (3.3)		
Admission to NICU	rate (%)	4 (6.7)	3 (4.9)	3 (4.9)	9 (15)		
Perinatal death	rate (%)	1 (1.7)	2 (3.3)	0	1 (1.6)		

Table shows rates and % of neonatal outcomes by quartiles of BMI change. Odds ratios are adjusted for periconceptional BMI, nulliparity and smoking. Composite neonatal outcome consisted of an abnormal cord pH (<7.05), Apgar <7 at 5 minutes, admission to the NICU and perinatal death (stillbirth above 24 weeks gestation or early neonatal death within six weeks postpartum). P-values for the linear relation of quartiles of BMI change were calculated using the quartiles as a continuous variable, with adjustment for confounders.

^a^ Women in Q1, Q2 and Q3 were grouped together in the analysis and compared to women in Q4

^b^ One woman with an ongoing pregnancy had no follow-up during pregnancy and outcomes were not recorded

^c^ The denominator is the number of live births (Q1 n = 59, Q2 n = 59, Q3 n = 62 and Q4 n = 61)

BMI, body-mass index, SGA, small-for-gestational age, aOR, adjusted odds ratio, CI, confidence interval, LGA, large-for-gestational age, NICU, neonatal intensive care unit

In addition, there were no significant differences in the rates of SGA, LGA or the composite adverse neonatal outcome among women within the weight change quartiles in kilograms (S2 Table).

### Subgroup analysis of singleton pregnancies

The results of the subgroup analysis on maternal and neonatal outcomes according to periconceptional change in BMI in singleton pregnancies are shown in S3 and S4 Tables. In this subgroup analysis, the significantly decreased rate of a hypertensive complication remained in women in Q2 (aOR 0.27, 95% CI 0.10–0.72) and Q3 (aOR 0.39, 95%CI 0.16–0.98) and when comparing women within Q1, Q2 and Q3 grouped together to women in Q4 (aOR 0.38, 95%CI 0.18–0.80). In addition, there was a significantly decreased rate of preterm birth in women in Q2 (aOR 0.24, 95% CI 0.06–0.98) compared to women in Q4 and when comparing women in Q1, Q2 and Q3 grouped together to women in Q4 (aOR 0.37, 95%CI 0.14–0.97). There were no significant differences in rates of excessive gestational weight gain in term pregnancies, gestational diabetes, induction of labor, spontaneous vaginal birth, assisted vaginal birth and Caesarean section between quartiles. The rates of LGA or SGA and the composite neonatal outcome were not different between quartiles.

### Effect modification of randomization group

The logistic regression analyses with addition of an interaction term for randomization group and the BMI change quartile did not show any statistically significant modification of the effect of BMI change by randomization group.

## Discussion

In this exploratory post-hoc analysis of pooled data of both treatment groups of the LIFEstyle RCT, we investigated the association between the magnitude of periconceptional BMI and weight change in quartiles and maternal and neonatal outcomes in obese infertile women. Our results show that women with a periconceptional BMI change between -2.1 and 0.1 kg/m^2^ (Q2 and Q3), had a decreased rate of hypertensive pregnancy complications compared to women who gained ≥0.1 kg/m^2^ during the periconceptional period. In addition, when women in Q1, Q2 and Q3 were grouped together and compared to women in Q4 there was an aOR of 0.39 (95%CI 0.19–0.82) of having a hypertensive complication during pregnancy. This was apparent in the total group and in the subgroup analysis including women with a singleton pregnancy. For women with a singleton pregnancy, the rate of preterm birth was significantly decreased in women who lost -2.1 to -0.9 kg/m^2^ (Q2) during the periconceptional period and when women in Q1, Q2 and Q3 were grouped together compared to women in Q4. There were no differences in rates of excessive gestational weight gain, gestational diabetes, induction of labor, mode of birth, small for gestational age or large for gestational age and the composite neonatal outcome among women in the different weight change quartiles.

Interestingly, women with the most periconceptional BMI and weight loss in kilograms (in Q1) did not have a significantly decreased risk of a hypertensive complication or preterm birth (in the subgroup analysis), while women in Q2 and/or Q3 periconceptional weight loss did have a significantly decreased risk of these complications. The point estimates of the adjusted odds ratios are well below 1 for all the BMI change quartiles, suggesting that the risk of these complications is lower after weight loss. The fact that this does not reach significance in all quartiles could be the consequence of the relatively small numbers of outcomes in these quartiles, which make the point estimates more susceptible to chance fluctuation. It is also possible that periconceptional BMI and weight loss (as seen in Q2 and Q3) instead of more substantial weight loss (as seen in Q1) is more favorable for decreasing complications, but there is no evidence supporting this hypothesis. The discrepancy in the incidence of preterm births between the main analysis and subgroup analysis can be explained by the higher risk of preterm birth usually present in multiple pregnancies [32].

A large body of evidence exists on the negative impact of increased maternal BMI on reproductive, maternal and neonatal outcomes. However, studies on the effects of lifestyle interventions to decrease periconceptional weight to possibly counteract the adverse effects of overweight are limited. This might be due to difficulties providing interventions for women who are planning a pregnancy, as they are generally healthy and have minimal engagement to the healthcare system. Furthermore, most studies focus on interpregnancy weight loss. Villamor and Cnattingius did not find a significant difference in the rate of gestational hypertension or gestational diabetes in overweight women who lost >1 kg/m^2^ before the next pregnancy, but there was a lower rate of LGA [26]. This was also observed by Jain et al., who found that obese women with an interpregnancy weight loss of ≥2 kg/m^2^ had an aOR 0.61 (95%CI 0.52–0.73) of having an LGA neonate [33]. We did not observe a decreased rate of LGA in obese women who achieved periconceptional weight loss. However, the rate of LGA was relatively low in our study. Our results are in agreement with findings of Mostello et al., who found that women who decreased their BMI between pregnancies were less likely to experience recurrent preeclampsia [34].

An explanation of the limited effects of lifestyle intervention on pregnancy outcomes in this study could be the limited amount of weight change that can be achieved through a lifestyle intervention. Studies that were performed to investigate the effects of preconception bariatric surgery, which usually results in a larger weight change, have shown it to be associated with decreased rates of gestational diabetes, hypertensive disorders, fetal macrosomia and LGA [21–23]. However, the risk of SGA is increased and some studies report a shorter duration of gestation [22,23]. Average weight loss that can be achieved by obese women of reproductive age after bariatric surgery is on average minus 8 to 15 kg/m^2^ [22]. This is much greater than weight loss that can be achieved with a lifestyle intervention with a duration of six months, as in our study. However, remarkably, the magnitude of effect in reduction of the rate of hypertensive disorders is comparable to that in our study. A biological explanation for this effect may lie in a reduction in low-grade inflammation after weight loss [34,35]. Obesity is associated with an increase in oxidative stress and inflammatory markers [36–38], as is seen in preeclampsia [39]. Weight loss is associated with improvement of this low-grade inflammation [35] and the decreased risk of hypertensive disorders could therefore be mediated through a decreased inflammatory state associated with weight loss [34].

### Strengths and limitations

The main strength of this study is that we used data from the largest prospective study so far on the effect of a preconception lifestyle intervention program targeted at weight loss in obese infertile women. By taking into account women all women who had an ongoing pregnancy in the LIFEstyle study we could create a large and unique cohort with prospectively collected periconceptional data on weight change and a subsequent pregnancy. In addition, due to the prospective data collection inherent to the RCT design of the LIFEstyle study, we were able to adjust for important confounders. While previous studies investigated relations between interpregnancy weight change and maternal or neonatal outcomes, our study provides a unique perspective, in which 77% of women are nulliparous.

A limitation of the current analysis is that the LIFEstyle study was powered on the healthy live birth rate within 24 months, instead of on adverse maternal and neonatal outcomes. Nevertheless, we were able to demonstrate some statistically significant associations between periconceptional weight loss and maternal outcomes. Secondly, since the cohort data we analyzed originate from a randomized controlled trial of a lifestyle intervention, effects of periconceptional weight loss on pregnancy outcomes could be confounded by effects of the lifestyle intervention other than weight loss. We found no evidence of such effects or of interaction between periconceptional weight change and treatment group. Even with these results confounding of the pregnancy outcomes by the lifestyle intervention cannot entirely be excluded and should be kept in mind when interpreting the results of this analysis. Finally, we were not able to control for all possible mediators of outcomes of pregnancy and/or periconceptional weight loss. Our results show that 56% of women gained excessive amounts of weight during pregnancy. Since only 54% of women with an ongoing pregnancy in our study provided the self-measured data of their weight change during pregnancy, we could not adjust our analyses for this factor. Excessive gestational weight gain is associated with an increased rate of various maternal and neonatal complications [40] and therefore could possibly mask the association between periconceptional weight change and outcomes of pregnancy. Currently, recording gestational weight gain and measures to prevent excessive gestational weight gain are not part of routine obstetric care in The Netherlands. We recommend that gestational weight gain should be recorded to facilitate research on outcomes of pregnancy in obese women. In addition, the amount of weight change an infertile woman can achieve before conception might be influenced by undergoing infertility treatment. Women who participated in the LIFEstyle study underwent different types of infertility treatment, therefore we could not construct reliable adjustments for this factor.

These data provide an insight into the possible magnitude of effect of periconceptional weight loss on perinatal outcomes. For the rare outcomes, especially in the neonate, combining our data with similar trials in an individual participant data meta-analysis could provide a more definite answer to the question whether periconceptional weight loss has a beneficial effect on maternal and neonatal outcomes in obese (infertile) women. Our estimates may be imprecise due to residual confounding, but their magnitude is clinically relevant and warrants further study.

## Conclusions

These results suggest that a periconceptional decrease in BMI in obese infertile women could lead to a decrease in the rate of a hypertensive pregnancy complication and preterm birth. Our study was not powered on adverse pregnancy outcomes and therefore these results should be seen as explorative, indicating the magnitude of effect on maternal and neonatal outcomes that can be achieved by a preconception weight loss program. However their magnitude is clinically relevant and warrants further study.

## Supporting information

S1 Table Maternal outcomes by quartile of periconceptional weight change in kg. (DOCX)

S2 Table Neonatal outcomes by quartile of periconceptional weight change in kg. (DOCX)

S3 Table Maternal outcomes by quartile of BMI change in singleton pregnancies. (DOCX)

S4 Table Neonatal outcomes by quartile of BMI change in singleton pregnancies. (DOCX)

S1 Dataset The dataset including relevant data in this paper. (SAV)

## References

[1] American College of Obstetricians and Gynecologists. ACOG Practice Bulletin No 156: Obesity in Pregnancy. Obstet Gynecol. 2015;126: e112–26. doi: 10.1097/AOG.0000000000001211 26595582 10.1097/AOG.0000000000001211

[2] Liu P, Xu L, Wang Y, Zhang Y, Du Y, Sun Y, et al Association between perinatal outcomes and maternal pre-pregnancy body mass index. Obes Rev. 2016;17: 1091–1102. doi: 10.1111/obr.12455 27536879 10.1111/obr.12455

[3] Eriksson JG, Sandboge S, Salonen M, Kajantie E, Osmond C. Maternal weight in pregnancy and offspring body composition in late adulthood: findings from the Helsinki Birth Cohort Study (HBCS). Ann Med. 2015;47: 94–99. doi: 10.3109/07853890.2015.1004360 25797690 10.3109/07853890.2015.1004360

[4] Gluckman PD, Hanson MA, Cooper C, Thornburg KL. Effect of in utero and early-life conditions on adult health and disease. N Engl J Med. 2008;359: 61–73. doi: 10.1056/NEJMra0708473 18596274 10.1056/NEJMra0708473 PMC3923653

[5] Ovesen P, Rasmussen S, Kesmodel U. Effect of prepregnancy maternal overweight and obesity on pregnancy outcome. Obstet Gynecol. 2011;118: 305–312. doi: 10.1097/AOG.0b013e3182245d49 21775846 10.1097/AOG.0b013e3182245d49

[6] Weiss JL, Malone FD, Emig D, Ball RH, Nyberg DA, Comstock CH, et al Obesity, obstetric complications and cesarean delivery rate—a population-based screening study. Am J Obstet Gynecol. 2004;190: 1091–1097. doi: 10.1016/j.ajog.2003.09.058 15118648 10.1016/j.ajog.2003.09.058

[7] Sebire NJ, Jolly M, Harris JP, Wadsworth J, Joffe M, Beard RW, et al Maternal obesity and pregnancy outcome: a study of 287,213 pregnancies in London. Int J Obes Relat Metab Disord. 2001;25: 1175–1182. doi: 10.1038/sj.ijo.0801670 11477502 10.1038/sj.ijo.0801670

[8] Denison FC, Norwood P, Bhattacharya S, Duffy A, Mahmood T, Morris C, et al Association between maternal body mass index during pregnancy, short-term morbidity, and increased health service costs: a population-based study. BJOG. 2014;121: 72–82. doi: 10.1111/1471-0528.12443 24102880 10.1111/1471-0528.12443

[9] Cnattingius S, Villamor E, Johansson S, Edstedt Bonamy AK, Persson M, Wikstrom AK, et al Maternal obesity and risk of preterm delivery. JAMA. 2013;309: 2362–2370. doi: 10.1001/jama.2013.6295 23757084 10.1001/jama.2013.6295

[10] Guelinckx I, Devlieger R, Beckers K, Vansant G. Maternal obesity: pregnancy complications, gestational weight gain and nutrition. Obesity Reviews. 2008;9: 140–150. doi: 10.1111/j.1467-789X.2007.00464.x 18221480 10.1111/j.1467-789X.2007.00464.x

[11] Stothard KJ, Tennant PW, Bell R, Rankin J. Maternal overweight and obesity and the risk of congenital anomalies: a systematic review and meta-analysis. JAMA. 2009;301: 636–650. doi: 10.1001/jama.2009.113 19211471 10.1001/jama.2009.113

[12] Aune D, Saugstad OD, Henriksen T, Tonstad S. Maternal body mass index and the risk of fetal death, stillbirth, and infant death: a systematic review and meta-analysis. JAMA. 2014;311: 1536–1546. doi: 10.1001/jama.2014.2269 24737366 10.1001/jama.2014.2269

[13] Yu Z, Han S, Zhu J, Sun X, Ji C, Guo X. Pre-pregnancy body mass index in relation to infant birth weight and offspring overweight/obesity: a systematic review and meta-analysis. PLoS One. 2013;8: e61627 doi: 10.1371/journal.pone.0061627 23613888 10.1371/journal.pone.0061627 PMC3628788

[14] Gademan MG, Vermeulen M, Oostvogels AJ, Roseboom TJ, Visscher TL, van Eijsden M, et al Maternal prepregancy BMI and lipid profile during early pregnancy are independently associated with offspring's body composition at age 5–6 years: the ABCD study. PLoS One. 2014;9: e94594 doi: 10.1371/journal.pone.0094594 24740157 10.1371/journal.pone.0094594 PMC3989215

[15] Reynolds RM, Allan KM, Raja EA, Bhattacharya S, McNeill G, Hannaford PC, et al Maternal obesity during pregnancy and premature mortality from cardiovascular event in adult offspring: follow-up of 1 323 275 person years. BMJ. 2013;347: f4539 doi: 10.1136/bmj.f4539 23943697 10.1136/bmj.f4539 PMC3805484

[16] Thangaratinam S, Rogozinska E, Jolly K, Glinkowski S, Roseboom T, Tomlinson JW, et al Effects of interventions in pregnancy on maternal weight and obstetric outcomes: meta-analysis of randomised evidence. BMJ. 2012;344: e2088 doi: 10.1136/bmj.e2088 22596383 10.1136/bmj.e2088 PMC3355191

[17] Dodd JM, Turnbull D, McPhee AJ, Deussen AR, Grivell RM, Yelland LN, et al Antenatal lifestyle advice for women who are overweight or obese: LIMIT randomised trial. BMJ. 2014;348: g1285 doi: 10.1136/bmj.g1285 24513442 10.1136/bmj.g1285 PMC3919179

[18] Muktabhant B, Lawrie TA, Lumbiganon P, Laopaiboon M. Diet or exercise, or both, for preventing excessive weight gain in pregnancy. Cochrane Database Syst Rev. 2015;(6):CD007145. doi: CD007145. doi: 10.1002/14651858.CD007145.pub3 26068707 10.1002/14651858.CD007145.pub3 PMC9428894

[19] Poston L, Bell R, Croker H, Flynn AC, Godfrey KM, Goff L, et al Effect of a behavioural intervention in obese pregnant women (the UPBEAT study): a multicentre, randomised controlled trial. Lancet Diabetes Endocrinol. 2015;3: 767–777. doi: 10.1016/S2213-8587(15)00227-2 26165396 10.1016/S2213-8587(15)00227-2

[20] Koivusalo SB, Rono K, Klemetti MM, Roine RP, Lindstrom J, Erkkola M, et al Gestational Diabetes Mellitus Can Be Prevented by Lifestyle Intervention: The Finnish Gestational Diabetes Prevention Study (RADIEL): A Randomized Controlled Trial. Diabetes Care. 2016;39: 24–30. doi: 10.2337/dc15-0511 26223239 10.2337/dc15-0511

[21] Maggard MA, Yermilov I, Li Z, Maglione M, Newberry S, Suttorp M, et al Pregnancy and fertility following bariatric surgery: a systematic review. JAMA. 2008;300: 2286–2296. doi: 10.1001/jama.2008.641 19017915 10.1001/jama.2008.641

[22] Yi XY, Li QF, Zhang J, Wang ZH. A meta-analysis of maternal and fetal outcomes of pregnancy after bariatric surgery. Int J Gynaecol Obstet. 2015;130: 3–9. doi: 10.1016/j.ijgo.2015.01.011 25863541 10.1016/j.ijgo.2015.01.011

[23] Johansson K, Cnattingius S, Naslund I, Roos N, Trolle Lagerros Y, Granath F, et al Outcomes of pregnancy after bariatric surgery. N Engl J Med. 2015;372: 814–824. doi: 10.1056/NEJMoa1405789 25714159 10.1056/NEJMoa1405789

[24] Mutsaerts MA, Groen H, ter Bogt NC, Bolster JH, Land JA, Bemelmans WJ, et al The LIFESTYLE study: costs and effects of a structured lifestyle program in overweight and obese subfertile women to reduce the need for fertility treatment and improve reproductive outcome. A randomised controlled trial. BMC Womens Health. 2010;10: 22-6874-10-22. 10.1186/1472-6874-10-22 PMC2907305 20579357

[25] Mutsaerts MA, van Oers AM, Groen H, Burggraaff JM, Kuchenbecker WK, Perquin DA, et al Randomized Trial of a Lifestyle Program in Obese Infertile Women. N Engl J Med. 2016;374: 1942–1953. doi: 10.1056/NEJMoa1505297 27192672 10.1056/NEJMoa1505297

[26] Villamor E, Cnattingius S. Interpregnancy weight change and risk of adverse pregnancy outcomes: a population-based study. Lancet. 2006;368: 1164–1170. doi: 10.1016/S0140-6736(06)69473-7 17011943 10.1016/S0140-6736(06)69473-7

[27] Dutch Society of Obstetrics and Gynaecology (NVOG). Guidelines subfertility. Available: http://nvog-documenten.nl/index.php?pagina=/richtlijn/pagina.php&fSelectTG_62=75&fSelectedSub=62&fSelectedParent=75.

[28] Institute of Medicine and National Research Council. Weight Gain During Pregnancy: Reexamining the Guidelines. Washington, DC: The National Academies Press; 2009. 20669500

[29] HAPO Study Cooperative Research Group, Metzger BE, Lowe LP, Dyer AR, Trimble ER, Chaovarindr U, et al Hyperglycemia and adverse pregnancy outcomes. N Engl J Med. 2008;358: 1991–2002. doi: 10.1056/NEJMoa0707943 18463375 10.1056/NEJMoa0707943

[30] Tranquilli AL, Dekker G, Magee L, Roberts J, Sibai BM, Steyn W, et al The classification, diagnosis and management of the hypertensive disorders of pregnancy: A revised statement from the ISSHP. Pregnancy Hypertension: An International Journal of Women's Cardiovascular Health. 2014;4: 97–104. 10.1016/j.preghy.2014.02.001 26104417

[31] Visser GH, Eilers PH, Elferink-Stinkens PM, Merkus HM, Wit JM. New Dutch reference curves for birthweight by gestational age. Early Hum Dev. 2009;85: 737–744. doi: 10.1016/j.earlhumdev.2009.09.008 19914013 10.1016/j.earlhumdev.2009.09.008

[32] Rydhstroem H, Heraib F. Gestational duration, and fetal and infant mortality for twins vs singletons. Twin Res. 2001;4: 227–231. 11665301 10.1375/1369052012434

[33] Jain AP, Gavard JA, Rice JJ, Catanzaro RB, Artal R, Hopkins SA. The impact of interpregnancy weight change on birthweight in obese women. Am J Obstet Gynecol. 2013;208: 205.e1–205.e7. 23246318 10.1016/j.ajog.2012.12.018

[34] Mostello D, Jen Chang J, Allen J, Luehr L, Shyken J, Leet T. Recurrent preeclampsia: the effect of weight change between pregnancies. Obstet Gynecol. 2010;116: 667–672. doi: 10.1097/AOG.0b013e3181ed74ea 20733450 10.1097/AOG.0b013e3181ed74ea

[35] Forsythe LK, Wallace JM, Livingstone MB. Obesity and inflammation: the effects of weight loss. Nutr Res Rev. 2008;21: 117–133. doi: 10.1017/S0954422408138732 19087366 10.1017/S0954422408138732

[36] Van Gaal LF, Mertens IL, De Block CE. Mechanisms linking obesity with cardiovascular disease. Nature. 2006;444: 875–880. doi: 10.1038/nature05487 17167476 10.1038/nature05487

[37] Heymsfield SB, Wadden TA. Mechanisms, Pathophysiology, and Management of Obesity. N Engl J Med. 2017;376: 254–266. doi: 10.1056/NEJMra1514009 28099824 10.1056/NEJMra1514009

[38] Kahn SE, Hull RL, Utzschneider KM. Mechanisms linking obesity to insulin resistance and type 2 diabetes. Nature. 2006;444: 840–846. doi: 10.1038/nature05482 17167471 10.1038/nature05482

[39] Walsh SW. Obesity: a risk factor for preeclampsia. Trends Endocrinol Metab. 2007;18: 365–370. doi: 10.1016/j.tem.2007.09.003 18023357 10.1016/j.tem.2007.09.003

[40] Nohr EA, Vaeth M, Baker JL, Sorensen TI, Olsen J, Rasmussen KM. Combined associations of prepregnancy body mass index and gestational weight gain with the outcome of pregnancy. Am J Clin Nutr. 2008;87: 1750–1759. 18541565 10.1093/ajcn/87.6.1750

