# Supplementary material for: Association between periconceptional weight loss and maternal and neonatal outcomes in obese infertile women
Source: PLoS One. 2018 Mar 28;13(3):e0192670. doi: 10.1371/journal.pone.0192670 (PMC5873932; doi:10.1371/journal.pone.0192670)
Supplement: S1 Table — (DOCX) [file pone.0192670.s001.docx]

|  | **Quartile** | **Q1** | **Q2** | **Q3** | **Q4** |  |  |
| --- | --- | --- | --- | --- | --- | --- | --- |
|  | ∆ kg | <-6.1 | -6.1 to -2.6 | -2.6 to 0.4 | >0.4 | aOR Q1to 3 vs Q4^a^ | P-value linear relation |
| **Rates of ongoing pregnancies within 24 months** | | | | | |  |  |
|  |  | n=61^b^ | n=60 | n=62 | n=61 |  |  |
| **Maternal outcomes** |  |  |  |  |  |  |  |
| Excessive gestational weight gain^c^ | rate (%) | 22/35 (63) | 20/30 (67) | 9/25 (36) | 14/27 (52) |  |  |
|  | aOR | 1.85 | 2.14 | 0.56 | 1.00 | 1.17 | 0.11 |
|  | (95%CI) | (0.55-6.22) | (0.61-7.52) | (0.17-1.85) |  | (0.43-3.24) |  |
| Gestational diabetes | rate (%) | 8 (13) | 8 (13) | 15 (24) | 10 (16) |  |  |
|  | aOR | 0.92 | 0.95 | 1.64 | 1.00 | 1.24 | 0.63 |
|  | (95%CI) | (0.29-2.90) | (0.32-2.85) | (0.63-4.27) |  | (0.52-2.99) |  |
| Hypertensive complications | rate (%) | 15 (25) | 7 (12) | 11 (18) | 21 (34) |  |  |
|  | aOR | 0.61 | 0.23 | 0.43 | 1.00 | 0.39 | 0.18 |
|  | (95%CI) | (0.24-1.53) | (0.08-0.64) | (0.18-1.03) |  | (0.19-0.82) |  |
| **Rates of live births conceived within 24 months** | | | | | | |  |
|  |  | n=59 | n=59 | n=62 | n=60 |  |  |
| Preterm birth | rate (%) | 8 (14) | 6 (10) | 5 (8.1) | 12 (20) |  |  |
|  | aOR | 0.64 | 0.45 | 0.36 | 1.00 | 0.46 | 0.49 |
|  | (95%CI) | (0.21-1.96) | (0.15-1.40) | (0.11-1.13) |  | (0.19-1.11) |  |
| Induction of labor | rate (%) | 25 (42) | 20 (34) | 25 (40) | 30 (50) |  |  |
|  | aOR | 0.91 | 0.61 | 0.73 | 1.00 | 0.73 | 0.75 |
|  | (95%CI) | (0.40-2.06) | (0.28-1.34) | (0.35-1.54) |  | (0.38-1.39) |  |
| Spontaneous vaginal birth | rate (%) | 33 (56) | 44 (75) | 36 (58) | 33 (55) |  |  |
|  | aOR | 0.78 | 2.04 | 0.93 | 1.00 | 1.15 | 1.0 |
|  | (95%CI) | (0.34-1.80) | (0.89-4.68) | (0.44-1.98) |  | (0.59-2.21) |  |
| Assisted vaginal birth^d^ | rate (%) | 8 (20) | 4 (8.3) | 13 (27) | 8 (20) |  |  |
|  | aOR | 1.63 | 0.47 | 2.04 | 1.00 | 1.25 | 0.86 |
|  | (95%CI) | (0.45-5.89) | (0.12-1.80) | (0.69-6.02) |  | (0.47-3.33) |  |
| Caesarean section | rate (%) | 18 (31) | 11 (19) | 13 (21) | 19 (32) |  |  |
|  | aOR | 1.03 | 0.52 | 0.61 | 1.00 | 0.67 | 0.97 |
|  | (95%CI) | (0.42-2.50) | (0.21-1.30) | (0.26-1.42) |  | (0.33-1.36) |  |

**S1 Table. Maternal outcomes by quartile of periconceptional weight change in kg**

Table shows rates and % of maternal outcomes by quartiles of periconceptional weight change in kilograms.

Odds ratios are adjusted for periconceptional BMI, nulliparity and smoking.

P-values for the linear relation of quartiles of BMI change were calculated using the quartiles as a continuous variable, with adjustment for confounders.

^a^ Women in Q1, Q2 and Q3 were grouped together in the analysis and compared to women in Q4

^b^ One woman with an ongoing pregnancy had no follow-up during pregnancy and outcomes were not recorded

^c^ In term pregnancies only

^d^ The denominator is the total number of vaginal births

BMI, body-mass index, aOR, adjusted odds ratio, CI, confidence interval
