# Supplementary material for: Association between periconceptional weight loss and maternal and neonatal outcomes in obese infertile women
Source: PLoS One. 2018 Mar 28;13(3):e0192670. doi: 10.1371/journal.pone.0192670 (PMC5873932; doi:10.1371/journal.pone.0192670)
Supplement: S2 Table — (DOCX) [file pone.0192670.s002.docx]

|  | **Quartile** | **Q1** | **Q2** | **Q3** | **Q4** |  |  |
| --- | --- | --- | --- | --- | --- | --- | --- |
|  | ∆ kg | <-6.1 | -6.1 to -2.6 | -2.6 to 0.4 | >0.4 | aOR Q1 to3 vs Q4^b^ | P-value linear relation |
|  |  | n=61^a^ | n=60 | n=62 | n=61 |  |  |
| **Maternal outcomes** |  |  |  |  |  |  |  |
| SGA^c^ | rate (%) | 5 (9) | 3 (5.1) | 4 (6.5) | 4 (6.7) |  |  |
|  | aOR | 1.01 | 0.58 | 0.89 | 1.00 | 0.80 | 0.93 |
|  | (95%CI) | (0.20-5.05) | (0.11-3.07) | (0.20-4.03) |  | (0.21-3.03) |  |
| LGA^c^ | rate (%) | 8 (14) | 11 (19) | 9 (15) | 9 (15) |  |  |
|  | aOR | 1.33 | 1.84 | 1.20 | 1.00 | 1.42 | 0.47 |
|  | (95%CI) | (0.42-4.19) | (0.64-5.25) | (0.42-3.41) |  | (0.58-3.50) |  |
| Composite neonatal outcome | rate (%) | 8 (13) | 5 (8.3) | 4 (6.5) | 10 (16) |  |  |
|  | aOR | 0.63 | 0.40 | 0.33 | 1.00 | 0.42 | 0.54 |
|  | (95%CI) | (0.19-2.08) | (0.12-1.37) | (0.10-1.15) |  | (0.16-1.12) |  |
| Abnormal cord pH | rate (%) | 1/40 (2.5) | 1/34 (2.9) | 1/38 (2.6) | 1/39 (2.6) |  |  |
| Apgar < 7 | rate (%) | 4 (6.7) | 2 (3.3) | 1 (1.6) | 2/60 (3.3) |  |  |
| Admission to NICU | rate (%) | 4 (6.7) | 3 (4.9) | 3 (4.9) | 9 (15) |  |  |
| Perinatal death | rate (%) | 1 (1.7) | 2 (3.3) | 0 | 1 (1.6) |  |  |

**S2 Table. Neonatal outcomes by quartile of periconceptional weight change in kg**

Table shows rates and % of neonatal outcomes by quartiles of periconceptional weight change. Odds ratios are adjusted for periconceptional BMI, nulliparity and smoking.

Composite neonatal outcome consisted of an abnormal cord pH (<7.05), Apgar <7 at 5 minutes, admission to the NICU and perinatal death (stillbirth above 24 weeks gestation or early neonatal death within six weeks postpartum).

P-values for the linear relation of quartiles of BMI change were calculated using the quartiles as a continuous variable, with adjustment for confounders.

^a^ One woman with an ongoing pregnancy had no follow-up during pregnancy and outcomes were not recorded

^b^ Women in Q1, Q2 and Q3 were grouped together in the analysis and compared to women in Q4

^c^ The denominator is the number of live births (Q1 n=59, Q2 n=59, Q3 n=62 and Q4 n=61)

BMI, body-mass index, SGA, small-for-gestational age, aOR, adjusted odds ratio, CI, confidence interval, LGA, large-for-gestational age, NICU, neonatal intensive care unit
