# Supplementary material for: Association between periconceptional weight loss and maternal and neonatal outcomes in obese infertile women
Source: PLoS One. 2018 Mar 28;13(3):e0192670. doi: 10.1371/journal.pone.0192670 (PMC5873932; doi:10.1371/journal.pone.0192670)
Supplement: S3 Table — (DOCX) [file pone.0192670.s003.docx]

# S3 Table. Maternal outcomes by quartile of BMI change in singleton pregnancies

|  | **Quartile** | **Q1** | **Q2** | **Q3** | **Q4** |  |  |
| --- | --- | --- | --- | --- | --- | --- | --- |
|  | ∆BMI | <-2.1 | -2.1 to -0.9 | -0.9 to 0.1 | >0.1 | aOR Q1 to3 vs Q4^a^ | P-value linear relation |
| **Rates of ongoing pregnancies within 24 months** | | | | | |  |  |
|  |  | n=56^b^ | n=60 | n=61 | n=59 |  |  |
| **Maternal outcomes** |  |  |  |  |  |  |  |
| Excessive gestational weight gain ^c^ | rate (%) | 21/34 (62) | 20/30 (67) | 10/26 (39) | 13/26 (50) |  |  |
|  | aOR | 1.82 | 2.42 | 0.65 | 1.00 | 1.25 | 0.13 |
|  | (95%CI) | (0.54-6.15) | (0.63-7.67) | (0.20-2.14) |  | (0.45-3.48) |  |
| Gestational diabetes | rate (%) | 8 (15) | 8 (13) | 15 (25) | 10 (17) |  |  |
|  | aOR | 1.01 | 0.91 | 1.63 | 1.00 | 1.24 | 0.71 |
|  | (95%CI) | (0.32-3.21) | (0.31-2.70) | (0.62-4.25) |  | (0.51-2.97) |  |
| Hypertensive complications | rate (%) | 13 (24) | 8 (13) | 10 (16) | 20 (34) |  |  |
|  | aOR | 0.55 | 0.27 | 0.39 | 1.00 | 0.38 | 0.15 |
|  | (95%CI) | (0.21-1.45) | (0.10-0.72) | (0.16-0.98) |  | (0.18-0.80) |  |
| **Rates of live births conceived within 24 months** | | | | | | |  |
|  |  | n=54 | n=59 | n=61 | n=58 |  |  |
| Preterm birth | rate (%) | 5 (9.3) | 3 (5.1) | 5 (8.2) | 11 (19) |  |  |
|  | aOR | 0.49 | 0.24 | 0.41 | 1.00 | 0.37 | 0.16 |
|  | (95%CI) | (0.14-1.79) | (0.06-0.98) | (0.13-1.34) |  | (0.14-0.97) |  |
| Induction of labour | rate (%) | 24 (44) | 20 (34) | 25 (41) | 29 (50) |  |  |
|  | aOR | 0.99 | 0.60 | 0.76 | 1.00 | 0.74 | 0.83 |
|  | (95%CI) | (0.43-2.30) | (0.27-1.32) | (0.36-1.61) |  | (0.38-1.43) |  |
| Spontaneous vaginal birth | rate (%) | 32 (59) | 44 (75) | 36 (59) | 33 (57) |  |  |
|  | aOR | 0.81 | 1.87 | 0.88 | 1.00 | 1.12 | 0.88 |
|  | (95%CI) | (0.34-1.91) | (0.81-4.28) | (0.41-1.90) |  | (0.57-2.18) |  |
| Assisted vaginal birth ^d^ | rate (%) | 7 (18) | 6 (12) | 12 (25) | 8 (20) |  |  |
|  | aOR | 1.43 | 0.69 | 1.88 | 1.00 | 1.25 | 0.87 |
|  | (95%CI) | (0.39-5.29) | (0.20-2.34) | (0.63-5.57) |  | (0.47-3.34) |  |
| Caesarean section | rate (%) | 15 (28) | 9 (15) | 13 (21) | 17 (29) |  |  |
|  | aOR | 1.05 | 0.47 | 0.71 | 1.00 | 0.68 | 0.87 |
|  | (95%CI) | (0.41-2.69) | (0.18-1.23) | (0.30-1.68) |  | (0.33-1.44) |  |

Table shows rates and % of maternal outcomes by quartiles of BMI change in women with an ongoing pregnancy.

Odds ratios are adjusted for periconception BMI, nulliparity and smoking.

P-values for the linear relation of quartiles of BMI change were calculated using the quartiles as a continuous variable, with adjustment for confounders

^a^ Women in Q1, Q2 and Q3 were grouped together in the analysis and compared to women in Q4

^b^ One woman with an ongoing pregnancy had no follow-up during pregnancy and outcomes were not recorded

^c^ In term pregnancies only

^d^ The denominator is the total number of vaginal births

BMI, body-mass index, aOR, adjusted odds ratio, CI, confidence interval
