# Supplementary material for: Association between periconceptional weight loss and maternal and neonatal outcomes in obese infertile women
Source: PLoS One. 2018 Mar 28;13(3):e0192670. doi: 10.1371/journal.pone.0192670 (PMC5873932; doi:10.1371/journal.pone.0192670)
Supplement: S4 Table — (DOCX) [file pone.0192670.s004.docx]

# S4 Table. Neonatal outcomes by quartile of BMI change in singleton pregnancies

|  | **Quartile** | **Q1** | **Q2** | **Q3** | **Q4** |  |  |
| --- | --- | --- | --- | --- | --- | --- | --- |
|  | ∆BMI | <-2.1 | -2.1 to -0.9 | -0.9 to 0.1 | >0.1 | aOR Q1 to 3 vs Q4^b^ | P-value linear relation |
|  |  | n=56^a^ | n=60 | n=61 | n=59 |  |  |
| **Maternal outcomes** |  |  |  |  |  |  |  |
| SGA^c^ | rate (%) | 4 (7.4) | 1 (1.7) | 4 (6.6) | 3 (5.2) |  |  |
|  | aOR | 0.94 | 0.23 | 1.09 | 1.00 | 0.73 | 0.68 |
|  | (95%CI) | (0.15-6.09) | (0.02-2.52) | (0.21-5.60) |  | (0.16-3.34) |  |
| LGA^c^ | rate (%) | 8 (15) | 10 (17) | 10 (16) | 8 (14) |  |  |
|  | aOR | 1.63 | 1.71 | 1.58 | 1.00 | 1.64 | 0.42 |
|  | (95%CI) | (0.50-5.35) | (0.58-5.05) | (0.55-4.58) |  | (0.65-4.16) |  |
| Composite neonatal outcome | rate (%) | 6 (11)^a^ | 4 (6.7) | 4 (6.6) | 9 (15) |  |  |
|  | aOR | 0.55 | 0.34 | 0.36 | 1.00 | 0.40 | 0.35 |
|  | (95%CI) | (0.15-2.00) | (0.09-1.29) | (0.10-1.32) |  | (0.14-1.11) |  |
| - Abnormal cord pH | rate (%) | 1 (1.8) | 1 (1.7) | 1 (1.6) | 1 (1.7) |  |  |
| - Apgar < 7 | rate (%) | 4 (7.3) | 2 (3.3) | 1 (1.6) | 2 (3.4) |  |  |
| - Admission to NICU | rate (%) | 2 (3.6) | 2 (3.3) | 3 (4.9) | 8 (14) |  |  |
| - Perinatal death | rate (%) | 1 (1.8) | 1 (1.7) | 0 | 1 (1.7) |  |  |

Table shows rates and % of neonatal outcomes by quartiles of BMI change in women with an ongoing pregnancy.

Odds ratios are adjusted for periconceptional BMI, nulliparity and smoking.

Composite neonatal outcome consisted of an abnormal cord pH (<7.05), Apgar <7 at 5 minutes, admission to the NICU and perinatal death (stillbirth above 24 weeks gestation or early neonatal death within six weeks postpartum).

P-values for the linear relation of quartiles of BMI change were calculated using the quartiles as a continuous variable, with adjustment for confounders

^a^ One woman with an ongoing pregnancy had no follow-up during pregnancy and outcomes were not recorded

^b^ Women in Q1, Q2 and Q3 were grouped together in the analysis and compared to women in Q4

^c^ The denominator is the number of live births

BMI, body-mass index, SGA, small-for-gestational age, aOR, adjusted odds ratio, CI, confidence interval, LGA, large-for-gestational age, NICU, neonatal intensive care unit
